# Enhanced cognitive-behavior therapy and family-based treatment for adolescents with an eating disorder: a non-randomized effectiveness trial

**DOI:** 10.1017/S0033291720004407

**Published:** 2022-10

**Authors:** Daniel Le Grange, Sarah Eckhardt, Riccardo Dalle Grave, Ross D. Crosby, Carol B. Peterson, Helene Keery, Julie Lesser, Carolyn Martell

**Affiliations:** 1 University of California, San Francisco, San Francisco, CA, USA; 2 The University of Chicago, Chicago, IL, USA (Emeritus); 3 Children's Minnesota, Minneapolis, St. Paul, MN, USA; 4 Villa Garda Hospital, Garda, Verona, Italy; 5 Sanford Center for Biobehavioral Research, Sanford Health, Fargo, ND, USA; 6 University of Minnesota, Minneapolis, MN, USA; 7 Rogers Behavioral Health, Minneapolis, MN, USA

**Keywords:** Enhanced cognitive-behavior therapy, family-based treatment, restrictive eating disorders, treatment effectiveness

## Abstract

**Background:**

Family-based treatment (FBT) is an efficacious intervention for adolescents with an eating disorder. Evaluated to a lesser degree among adolescents, enhanced cognitive-behavior therapy (CBT-E) has shown promising results. This study compared the relative effectiveness of FBT and CBT-E, and as per manualized CBT-E, the sample was divided into a *lower weight* [<90% median body mass index (mBMI)], and *higher weight* cohort (⩾90%mBMI).

**Method:**

Participants (*N* = 97) aged 12–18 years, with a DSM-5 eating disorder diagnosis (largely restrictive, excluding Avoidant Restrictive Food Intake Disorder), and their parents, chose between FBT and CBT-E. Assessments were administered at baseline, end-of-treatment (EOT), and follow-up (6 and 12 months). Treatment comprised of 20 sessions over 6 months, except for the *lower weight* cohort where CBT-E comprised 40 sessions over 9–12 months. Primary outcomes were slope of weight gain and change in Eating Disorder Examination (EDE) Global Score at EOT.

**Results:**

Slope of weight gain at EOT was significantly higher for FBT than for CBT-E (*lower weight*, est. = 0.597, s.e. = 0.096, *p* < 0.001; *higher weight*, est. = 0.495, s.e. = 0.83, *p* < 0.001), but not at follow-up. There were no differences in the EDE Global Score or most secondary outcome measures at any time-point. Several baseline variables emerged as potential treatment effect moderators at EOT. Choosing between FBT and CBT-E resulted in older and less well participants opting for CBT-E.

**Conclusions:**

Results underscore the efficiency of FBT to facilitate weight gain among underweight adolescents. FBT and CBT-E achieved similar outcomes in other domains assessed, making CBT-E a viable treatment for adolescents with an eating disorder.

**Clinical Trial Registration Information:**

Treatment Outcome in Eating Disorders; https://clinicaltrials.gov/; NCT03599921.

## Introduction

Eating disorders are life-threatening and highly prevalent psychiatric disorders that have a profound impact on the well-being of sufferers and their families (Swanson, Crow, Le Grange, Swendsen, & Merikangas, 2011). These disorders are associated with several psychiatric and medical morbidities which result in impairment in psychological as well as physiological domains (American Psychiatric Association, 2013). Treatment outcomes for these disorders remain uncertain, and for many patients, the illness continues to run a relapsing and unremitting course (Wonderlich, Bulik, Schmidt, Steiger, & Hoek, 2020).

Treatment outcome data for adolescents across the spectrum of eating disorder diagnoses remain modest. Notwithstanding, some promising treatment modalities for this patient population have emerged. Family-based treatment (FBT) has been evaluated in several randomized clinical trials (RCTs) for anorexia nervosa (AN) (c.f., Le Grange et al., 2016; Lock et al., 2010; Madden et al., 2015), as well as for bulimia nervosa (BN) (Le Grange, Crosby, Rathouz, & Leventhal, 2007; Le Grange, Lock, Agras, Bryson, & Jo, 2015). These RCTs have demonstrated that FBT is an efficacious intervention for this patient population, and could be considered the current first-line approach for those patients who are medically fit for outpatient management (Lock & Le Grange, 2019). That said, recent cohort studies (Dalle Grave, Calugi, Doll, & Fairburn, 2013; Dalle Grave, Calugi, Sartirana, & Fairburn, 2015; Dalle Grave, Sartirana, & Calugi, 2019b) have demonstrated that a version of enhanced cognitive-behavioral therapy (CBT-E), adapted for adolescents with an eating disorder, is a promising transdiagnostic intervention for this patient population. Also relatively unexplored are potential moderators of treatment outcome for adolescents with eating disorders (c.f. Le Grange et al., 2012, 2015, 2016; Lock, Agras, Bryson, & Kraemer, 2005; Madden et al., 2015). While findings from such studies are hypothesis generating at this time, they do suggest that eating-related obsessionality and eating disorder-specific psychopathology are likely candidates as moderators of treatment outcome.

The efficacy of CBT-E for adult eating disorders is well established (Byrne et al., 2017; Poulsen et al., 2014), and as described above, has been adapted for an adolescent patient population (Dalle Grave & Calugi, 2020) with promising outcomes (Dalle Grave et al., 2013). An obvious next step, and the primary goal of the present study, was to compare the relative effectiveness of FBT and CBT-E on measures of weight and eating disorder symptomatology among adolescents presenting with a DSM-5 eating disorder diagnosis [excluding Avoidant/ Restrictive Food Intake Disorder (ARFID)]. A secondary goal was to conduct an exploratory moderator analysis to investigate which of these two treatments might be more optimal for different patient groups. In this effectiveness design, families were given a choice between FBT and CBT-E. Therefore, a tertiary goal was to learn whether this choice might result in differences between treatment groups along key clinical parameters.

## Method

**Fig. 1.**
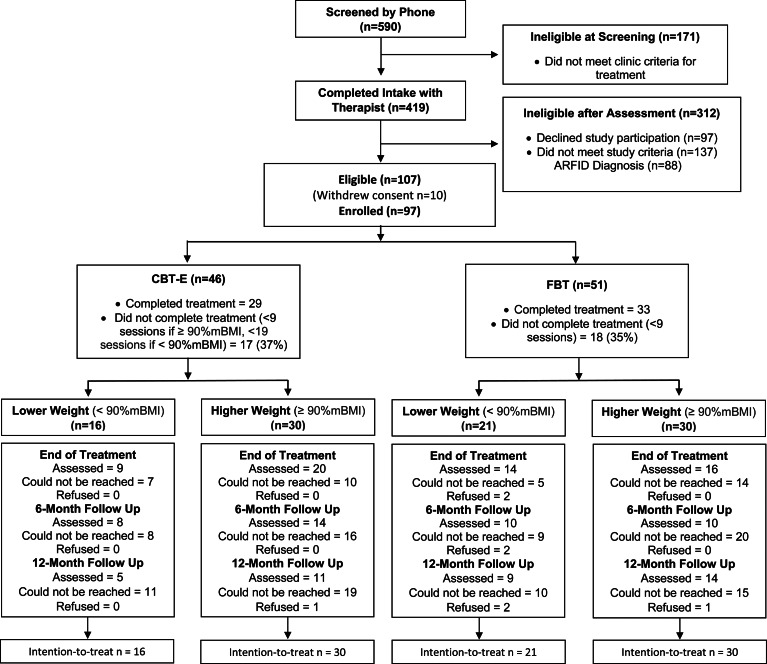
Consolidated standards of reporting trials (CONSORT) diagram. *Note*: FBT, family-based treatment; CBT-E, enhanced cognitive-behavior therapy.

The Center for the Treatment of Eating Disorders (CTED) at Children's Minnesota, MN, a pediatric specialty clinic in the USA, provides inpatient and outpatient treatment to youth and their families. This program provides care to an average number of about 400 patients per year for medical stabilization and/or outpatient management. Over the course of the study period (July 2015–November 2019), 419 patients completed an intake assessment. Of these, 312 were ineligible; 97 declined to participate in research, 137 did not meet study criteria (i.e. prior FBT/CBT-E; did not commit to treatment; >19 years; no eating disorder diagnosis; no interpreter in native language; substance dependency; co-existing medical diagnosis), and 88 met criteria for ARFID. Consequently, 107 patients met the eligibility criteria for the study. Of those, 10 families withdrew consent, and 97 patients (83%) and their families were enrolled and offered a choice between one of two manualized treatments: FBT (Le Grange & Lock, 2007; Lock & Le Grange, 2012) or CBT-E (Dalle Grave & Calugi, 2020). Fifty-one (52.5%) participants/families chose FBT, and 46 (47.5%) chose CBT-E (see Fig. 1). Because the content and duration of CBT-E differ based on the adolescent's initial weight, the sample was divided into a *lower weight* cohort [<90% median body mass index (mBMI); 38% of participants), and a *higher weight* cohort (⩾90%mBMI; 62% of participants).

### Participants

All patients were approached by a research assistant, who explained treatment and research procedures. At intake, and prior to the start of treatment, consent (parents and participants ⩾18 years of age) and assent (participants ⩽17 years of age) were obtained. Participants included youth aged 12–19 years old, living with their families/guardian, planning to engage in outpatient treatment at CTED for a DSM-5 diagnosed eating disorder (excluding ARFID), and medically stable for outpatient treatment. Participants were excluded when diagnosed with a co-morbid medical disorder known to influence eating or weight (i.e. pregnancy, cancer); psychotic disorder; acute suicidality; or substance abuse and/or substance dependence.

### Assessments and procedures

Intake consists of a comprehensive multi-disciplinary assessment, and involves the following meetings: (1) medical assistant to assess vital sign stability, reviewed by a psychiatrist (Society of Adolescent Medicine guidelines; Golden et al., 2015); (2) research staff/CTED clinician for semi-structured diagnostic interviews (Eating Disorder Examination (EDE) (Fairburn, Cooper, & O'Connor, 2008), and a battery of questionnaires; (3) diagnostic assessment with a clinician; and (4) consultation with a psychiatrist to review care plan. Patients, whose first contact with CTED was in the inpatient medical setting, completed the diagnostic interviews and battery of questionnaires before discharge to outpatient care. Given that hospital stays were quite brief (M = 13.1 days, s.d. = 10), assessments were not repeated upon starting outpatient treatment. This study was approved by the Institutional Review Board of Children's Minnesota, MN.

### Measures and semi-structured diagnostic interviews

Primary outcomes were; (1) rate of weight gain (%mBMI) and (2) eating disorder psychopathology [EDE or EDE-Questionnaire (EDE-Q) (Fairburn & Beglin, 1994)] from baseline to end-of-treatment (EOT) [The EDE-Q was utilized when the EDE was not available given the high concordance rates between these two measures (c.f. Berg et al., 2012)]. Semi-structured interviews as well as paper-and-pencil measures were completed with adolescents and parents at baseline, EOT, and at 6 and 12 months posttreatment. No remuneration for completion of assessments was available to participants.

Secondary outcomes were the Clinical Impairment Assessment (CIA) (Bohn & Fairburn, 2008), Beck Anxiety Inventory (BAI) (Beck & Steer, 1993), Child Depression Inventory (CDI-2) (Kovacs, 2010), and the Rosenberg Self-Esteem Scale (RSE) (Rosenberg, 1965). Parents completed the Child Behavior Checklist (CBCL) (Achenbach & Edelbrock, 1983), the Brief Symptom Inventory (BSI) (Derogatis & Spencer, 1993), and the McMaster Family Assessment Device (FAD) (Epstein, Baldwin, & Bishop, 1983). The Mini International Neuropsychiatric Interview for Children and Adolescents (MINI-Kid) (Sheehan et al., 2010) was used to assess co-occurring psychiatric diagnoses, and was conducted at baseline only. At weeks 2, 4, 6, 8, 10, and 12 of treatment, patients rated the suitability of their treatment, and how successful they thought the therapy would be, on a 10-point Likert scale.

### Treatments

In this non-randomized effectiveness study, patients and their parents were given a choice between FBT (Le Grange & Lock, 2007; Lock & Le Grange, 2012) or CBT-E (Dalle Grave & Calugi, 2020; Fairburn, 2008). At the initial assessment, families were provided two one-page information sheets, one for FBT and one for CBT-E, which described each treatment in terms of patient and family expectations should they select one treatment as opposed to the other (see Supplemental Materials, and for a description of the conceptual distinctions between FBT and CBT-E, see Dalle Grave, et al. (2019a)). All treating clinicians (doctoral-level psychologists and masters-level clinical social workers) received in-person training in FBT and CBT-E before the start of the study, and followed by monthly supervision for the duration of the study. Training and supervision were delivered by expert developers of these treatments.

*Family-Based Treatment* (Le Grange & Lock, 2007; Lock & Le Grange, 2012). FBT for adolescent eating disorders usually includes all members of the adolescent's immediate family. Treatment progresses through three phases, with the first (~10 sessions) focusing mainly on guiding the parents to support their adolescent toward weight restoration (when appropriate), and disrupting eating disorder behaviors (e.g. binge eating and purging). The second phase (~5–7 sessions) focuses on assisting the parents to restore food choices to the adolescent, with an emphasis on the developmental stage of the adolescent. Phase 3 is brief (2–3 sessions), focusing on adolescent developmental matters and helping the parents and their offspring navigate these tasks largely in the absence of acute eating disorder symptoms. Twenty treatment sessions are provided over a span of approximately 6 months.

*Enhanced Cognitive-Behavior Therapy* (Dalle Grave & Calugi, 2020; Fairburn, 2008). CBT-E is designed to address the core psychopathology of all eating disorders. CBT-E posits the eating problem as belonging to the individual, and is designed to encourage the adolescent, rather than their parent, to take control of the problem. Parents are not excluded from participating in treatment, but their involvement is limited to helping create a family environment that allows for recovery (c.f., Dalle Grave et al., 2019a). Patients are actively involved in all phases of treatment, including the decision to address weight regain and/or binge eating and purging, with the goal of promoting self-management. CBT-E is a collaborative approach, and patients are encouraged to be active in making changes. A primary goal of CBT-E is to address the patient's eating disorder psychopathology, i.e. patients' concerns about shape, weight, dietary restraint and restriction, and other extreme weight control behaviors. Following manualized CBT-E guidelines, for patients in the *lower weight* cohort, treatment involves 40 sessions over 9–12 months. For those in the *higher weight* cohort, treatment involves 20 sessions over the course of 6 months.

### Participant safety and hospitalization criteria

Participants were followed by their primary care physician/CTED psychiatrist to ensure medical stability throughout the study. If unstable, or presenting with an acute psychiatric risk, participants were admitted to the inpatient unit at Children's Minnesota, MN, but were not dropped from the study as a result of such required inpatient care.

### Statistical analysis

No *a priori* power analysis was conducted as our goal for this effectiveness study was to enroll the maximum number of eligible patients given our timeframe. Based upon the differing length of treatment in manualized CBT-E, which is based upon baseline weight status, all analyses were stratified by weight at beginning of treatment. This resulted in a *lower weight cohort*, which included patients <90%mBMI, and a *higher weight cohort*, which included patients ⩾90%mBMI. FBT and CBT-E were compared at baseline on demographic and clinical characteristics using χ^2^ or Fisher's exact tests for categorical data and independent *t* tests or Mann–Whitney *U* non-parametric tests for continuous measures.

Mixed-effects linear models were used to compare the trajectory of %mBMI between FBT and CBT-E across treatment visits and follow-up assessments. Analyses were based upon all available data and missing data were not imputed. Models included random intercepts, and fixed effects for treatment group, study visit^1^, and treatment group-by-study visit interactions. Fixed effects for quadratic (study visit) and cubic (study visit) components were added to allow non-linear trajectories. Model estimation was based upon full information maximum likelihood. Given the non-randomized nature of the design, baseline measurements were included as data points in the model rather than as covariates. This allowed for a comparison between treatment groups at baseline. Moderators of treatment were tested by adding main effects for moderator, as well as interactions for moderator-by-study visit, moderator-by-treatment group, and moderator-by-study visit-by-treatment group. Sensitivity analyses were conducted adjusting the analyses for propensity score, which represented the predicted probability of choosing FBT using all available baseline demographic and clinical assessments. Logistic regression analyses were conducted using all available baseline and demographic characteristics to predict the treatment selected (CBT-E or FBT). The predicted probability of selecting CBT-E for each participant, referred to as a propensity score, was derived from this model. Sensitivity analyses were conducted for all primary and secondary outcomes using propensity score as a covariate in the model.

Mixed-effects repeated-measures models were used to compare treatment groups on EDE/EDE-Q scores and secondary outcome measures at baseline, EOT, 6- and 12-month follow-up. Again, models were based upon available data and missing data were not imputed. Models included a random intercept, and fixed effects for treatment group, study visit, and treatment group-by-study visit interactions. *Post hoc* contrasts were used to compare treatment groups at each study visit.

## Results

### Study participants

Participants (*N* = 97) presented with an average age of 14.6 years (s.d. = 1.8, range = 11–19), mean duration of illness of 17.0 months (s.d. = 18.3, range = 1–84), and were mostly female (80 of 97; 82.5%). The mean percent mBMI for the *lower weight* cohort (*N* = 37) was 83.6 (s.d. = 4.1, median = 83.4, range = 74.8–89.8), and 103.7 (s.d. = 11.9, median = 101.21, range = 90.3–142.1) for the *higher weight* cohort. The majority of participants met DSM-5 criteria for AN or AAN (76 of 97; 78%), for the *lower weight* cohort, 92% met criteria for AN [the remainder met criteria for atypical AN (AAN) or UFED], and for the *higher weight* cohort, 67% met criteria for AN^2^ (*n* = 12) or AAN (*n* = 28), 20% BN, purging disorder or BED, and 13% UFED (the majority presenting with body image concerns, but without significant weight loss to meet the criteria for AAN). Weight gain was a primary treatment goal for the majority of this study sample (84 of 97; 87%). Most participants identified as Caucasian [86 of 97; 89% (Hispanic = 9)], and the remainder as African American (1 of 97; 1%), Asian (3 of 97; 3.1%), Multiracial/Other (4 of 97; 4.1%), and Not reported (3 of 97; 3.1%), and most participants lived with their family of origin (67 of 97; 69.1%). More than two-thirds had a history of prior mental health treatment and/or psychiatric hospitalization (67 of 97; 69.1%). Table 1 presents baseline demographic and clinical characteristics dividing the sample into the two weight cohorts by treatment modality. Regardless of weight cohort, participants who elected CBT-E were older, had been ill longer, presented with higher depression and anxiety, more prior mental health treatment, and higher rates of psychosocial impairment due to eating disorder features (all *p*s 0.034–0.0001).

**Table 1.** Participant characteristics FBT *v*. CBT-E by weight status

	FBT (*N* = 51)		CBT-E (*N* = 46)		FBT *v*. CBT-E Lower Wt	FBT *v*. CBT-E Higher Wt
	Lower Wt (*n* = 21)	Higher Wt (*n* = 30)	Lower Wt (*n* = 16)	Higher Wt (*n* = 30)		
Age (M/s.d.)	13.62 (1.83)	13.73 (1.51)	15.88 (1.54)	15.4 (1.4)	**<0.001**	**<0.001**
Weight (%mBMI)	82.25 (3.97)	102.29 (12.09)	85.42 (3.50)	105.15 (11.72)	**0.015**	0.356
Duration ill (mo)	11.18 (11.92)	9.61 (6.37)	28.93 (20.57)	22.04 (23.62)	**0.003**	**0.010**
Female (%)	86	73	94	83	0.43	0.266
Caucasian (Hisp + Non-Hisp) (%)^a^	86	87	88	93	0.333	0.149
DSM-5 AN/AAN (%)^b^	95	67	100	57	0.376	0.373
DSM-5 Co-occurring (%)	52	43	63	70	0.391	**0.034**
Previous MH Tx (%)	29	43	69	73	**0.017**	**0.018**
Previous Psych Hosp (%)	14	3	25	23	0.342	**0.026**
Medical Dx (%)	5	3	0	3	0.568	0.177
Parents married (%)	76	73	63	63	0.294	0.290
Hx any abuse (%)	5	0	19	10	0.206	0.119
Substance abuse (%)	0	0	0	3	^c^	0.500
BAI (M/s.d.)	15.44 (10.74)	10.81 (8.58)	19.13 (12.95)	18.86 (12.69)	0.372	**0.008**
CIA (M/s.d.)	17.53 (12.41)	11.12 (9.96)	29.0 (11.45)	25.53 (12.94)	**0.010**	**<0.001**
CDI-2 (M/s.d.)	55.43 (13.67)	56.97 (15.09)	64.5 (11.41)	66.44 (13.28)	**0.048**	**0.018**
RSE (M/s.d.)	18.89 (7.88)	19.41 (7.40)	11.63 (5.48)	11.53 (5.48)	**0.004**	**<0.001**
BSI (M/s.d.)	50.74 (11.40)	48.00 (13.35)	49.25 (10.56)	51.46 (11.72)	0.719	0.333
CBCL total (M/s.d.)	54.74 (12.14)	54.82 (10.62)	57.0 (9.43)	59.08 (10.79)	0.557	0.150
FAD (M/s.d.)	18.81 (4.29)	21.62 (5.43)	24.13 (4.47)	23.89 (6.02)	**0.001**	0.144

BAI, Beck Anxiety Inventory; CIA, Clinical Impairment Assessment; CDI-2, Child Depression Inventory; CBCL, Child Behavior Checklist; FAD, Family Assessment Device.

a Percent Caucasian (Hispanic + non-Hispanic).

b DSM-5 AN or AAN = 76; BN, BED or Purging Disorder = 12; UFED = 9.

c χ^2^ value not calculable (zero cases).

Bold p-values = statistical significance.

### Treatment completion

Among the *lower weight* FBT cohort, five (23.8%) participants completed <50% of treatment sessions, seven (33.3%) completed 50–75% of sessions, and nine (42.9%) completed >75% of sessions. Among the *lower weight* CBT-E cohort, five (31.3%) participants completed <50% of treatment sessions, five (31.3%) completed 50–75% of sessions, and six (37.5%) completed >75% of sessions (χ^2^ = 0.26, df = 2, *p* = 0.877). In the *higher weight* FBT cohort, 15 (50.0%) participants completed <50% of treatment sessions, three (10.0%) completed 50–75% of sessions, and 12 (40.0%) completed >75% of sessions. Among the *higher weight* CBT-E cohort, two (6.7%) completed <50% of treatment sessions, six (20.0%) completed 50–75% sessions, and 22 (73.3%) completed >75% of sessions (χ^2^ = 13.88, df = 2, *p* = 0.001).

### Primary and secondary treatment outcomes

Primary and secondary outcomes at each time point across the two treatment groups were initially conducted without adjustment. Results of sensitivity analyses using propensity adjustment produced comparable results to unadjusted models. Consequently, we only present the unadjusted results here.

**Fig. 2.**
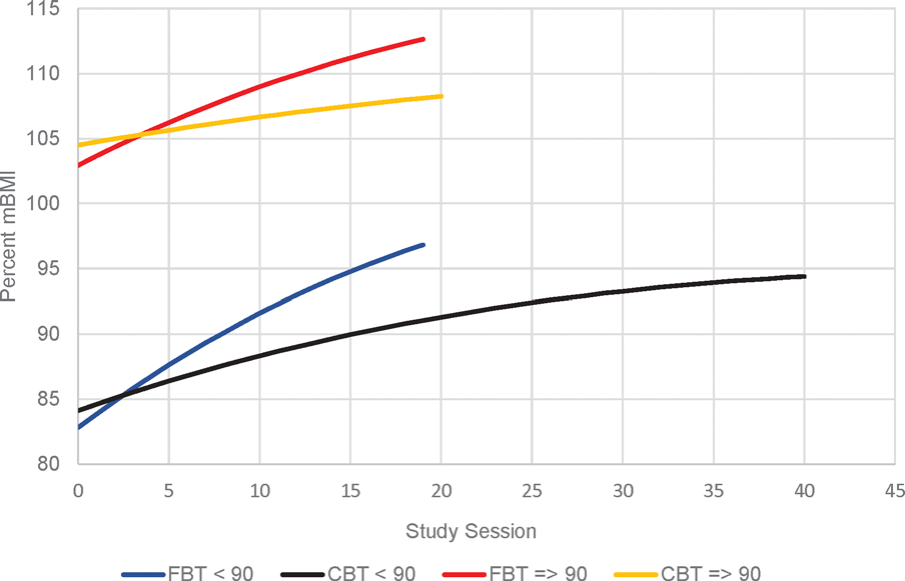
Slope of weight gain (percent mBMI) for FBT *v*. CBT-E at EOT.

**Fig. 3.**
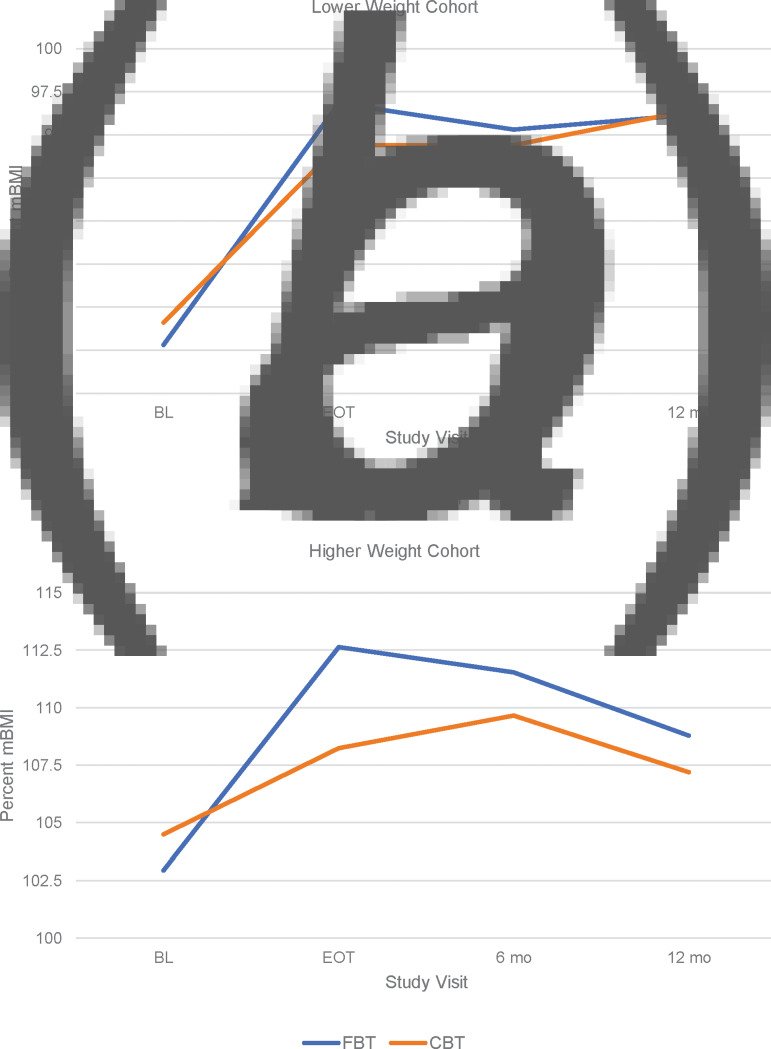
Slope of weight (% mBMI) for FBT *v*. CBT-E baseline to follow-up.

Figure 2 presents the primary outcome of weight gain in the lower weight and higher weight cohorts. There were no differences between FBT and CBT-E in weight (%mBMI) at the beginning of treatment in either the *lower weight* cohort (est. = −2.361, s.e. = 1.854, *p* = 0.207) or the *higher weight* cohort (est. = −2.283, s.e. = 2.994, *p* = 0.449). However, the slope of weight gain at EOT was significantly higher for FBT than for CBT-E in the *lower weight* cohort (est. = 0.597, s.e. = 0.096, *p* < 0.001) and the *higher weight* cohort (est. = 0.495, s.e. = 0.83, *p* < 0.001) (Fig. 2). Table 2 presents the average %mBMI by weight cohort at baseline, end of treatment and 6- and 12-month follow-up. There were no differences in weight gain between treatment groups, across weight cohorts, at the 6- or 12-month follow-up time points (Fig. 3a and 3b). There were no significant differences between FBT and CBT-E at EOT and at follow-up on eating disorder symptomatology (EDE/Q Global Score) in either weight cohort.

**Table 2.** Secondary outcomes: baseline through 12-month follow-up (M/s.d.)

	FBT (*N* = 51)								CBT-E (*N* = 46)								Sign
	FBT lower weight (*n* = 21)				FBT higher weight (*n* = 30)				CBT-E lower weight (*n* = 16)				CBT-E higher weight (*n* = 30)				
	B	EOT	6 m	12 m	B	EOT	6 m	12 m	B	EOT	6 m	12 m	B	EOT	6 m	12 m	
Primary outcome																	
%mBMI	82.8	**96.8**^a^	95.3	96.2	102.9	**112.6**^b^	111.5	108.8	84.1	**94.4**^a^	94.4	96.4	104.5	**108.3**^b^	109.7	107.2	***p* < 0.001**^a^
																	***p* < 0.001**^b^
EDE/Q-G	1.9	0.9	0.7	0.5	1.5	0.2	0.1	0.3	3.3	1.2	1.3	0.8	3.1	1.2	1.1	1.5	NS
	*1.5*	*1.4*	*1.2*	*0.9*	1.3	0.3	0.2	0.3	*1.1*	*1.0*	*1.4*	*0.4*	*1.5*	*1.5*	*1.2*	*1.7*	NS
Secondary outcomes																	
BAI	15.4	12.4	5.3	8.9	**10.8**^a^	**3.9**^b^	5.1	4.4	19.1	7.4	7.9	6.0	**18.9**^a^	**12.7**^b^	12.6	12.4	NS
	*10.7*	*11.2*	*7.2*	*11.1*	8.6	4.8	6.0	4.0	*12.9*	*5.7*	*6.5*	*4.4*	*12.7*	*12.7*	*12.3*	*13.0*	**ME**^a,b^
CIA	17.5	7.1	5.5	4.9	**11.1**^a^	2.1	2.7	6.2	29.0	7.5	7.9	3.8	**25.5**^a^	7.1	9.0	12.1	NS
	*12.4*	*9.4*	*10.1*	*8.7*	10.0	4.4	5.5	8.2	*11.4*	*7.3*	*7.8*	*2.6*	*12.9*	*9.6*	*11.0*	*15.9*	**ME**^a^
CDI-2	55.4	50.3	46.6	47.0	**57.0**^a^	47.1	46.6	**44.5**^c^	64.5	48.9	56.3	51.0	**66.4**^a^	52.1	54.2	**58.4**^c^	NS
	*13.7*	*9.2*	*8.0*	*10.1*	15.1	6.8	7.3	6.2	*11.4*	*5.5*	*16.4*	*10.9*	*13.3*	*10.4*	*11.0*	*16.9*	**ME**^a,c^
BSI	50.7	49.0	45.1	48.7	48.0	49.2	46.0	43.3	49.3	48.1	43.5	42.8	51.5	50.1	52.0	46.7	NS
	*11.4*	*9.0*	*11.3*	*11.4*	*13.4*	*11.6*	*11.9*	*11.1*	*10.6*	*7.7*	*6.8*	*6.9*	*11.7*	*11.2*	*11.4*	*8.5*	NS
RSE	18.9	20.4	22.1	19.9	**19.4**^a^	**24.1**^b^	**25.2**^d^	20.3	11.6	18.9	19.4	19.5	**11.5**^a^	**19.9**^b^	**17.3**^d^	16.5	NS
	*7.9*	*6.1*	*6.2*	*8.8*	*7.4*	*4.5*	*5.7*	*9.4*	*5.5*	*4.7*	*7.2*	*8.5*	*5.5*	*6.0*	*7.0*	*5.3*	**ME**^a,b,d^
FAD	18.8	19.7	19.3	18.1	21.6	21.7	21.3	20.7	24.1	20.1	24.8	18.3	23.9	22.8	21.9	23.4	NS
	*4.3*	*5.8*	*4.6*	*3.3*	*5.4*	*6.4*	*6.3*	*5.0*	*4.5*	*5.7*	*7.2*	*2.1*	*6.0*	*4.7*	*4.9*	*2.6*	NS
CBCLT	54.7	50.1	42.9	48.0	54.8	45.2	45.4	**43.7**^c^	57.0	46.9	48.3	48.8	59.1	50.9	49.0	**53.4**^c^	NS
	*12.1*	*9.9*	*11.0*	*12.6*	10.6	13.1	11.0	10.8	*9.4*	*9.7*	*11.5*	11.0	*10.8*	*11.7*	*14.1*	*9.1*	**ME**^c^

EDE/Q-G, Eating Disorder Examination (or Questionnaire) Global Score; BAI, Beck Anxiety Inventory; CIA, Clinical Impairment Assessment; CDI-2, Child Depression Inventory; BSI, Brief Symptom Inventory; RSE, Rosenberg Self-Esteem Scale; FAD, Family Assessment Device; CBCLT, Child Behavior Checklist Total Score.

*Key:* Primary outcome = slope of weight gain (%mBMI) and main effect for treatment (EDE/Q) at EOT; ME = main effect; ME refers to comparison between FBT and CBT-E at each time point, i.e.: ^a^Baseline (B) comparison of %mBMI between treatments, separately by weight cohort; ^b^EOT comparison; ^c^12-month follow-up (12 m) comparison; ^d^6-month follow-up (6 m) comparison.

Int = interaction effect, that is, treatment by visit interaction; and NS = no significant ME or interaction; superscript refers to ME at time point, e.g. baseline, EOT, etc.

Bold p-values = statistical significance.

As for secondary outcomes (Table 2), there were significant differences between FBT and CBT-E at all time points, favoring FBT. However, these were likely due to significant baseline differences between treatment groups in variables of interest. The only exceptions were for the *higher weight* cohort where CBCL (internalizing) was significantly lower for FBT than CBT-E at EOT (50.1 *v.* 58.7; est. = −8.73, s.e. = 3.66, *p* = 0.019), and at 12-month follow-up (49.6 *v.* 61.3; est. = 12.00, s.e. = 4.42, *p* = 0.008), and CBCL-T was significantly lower for FBT than CBT-E at 12-month follow-up (43.7 *v.* 53.4; est. = −9.22, s.e. = 4.42, *p* = 0.040).

### Treatment effect moderators of primary outcome

**Fig. 4.**
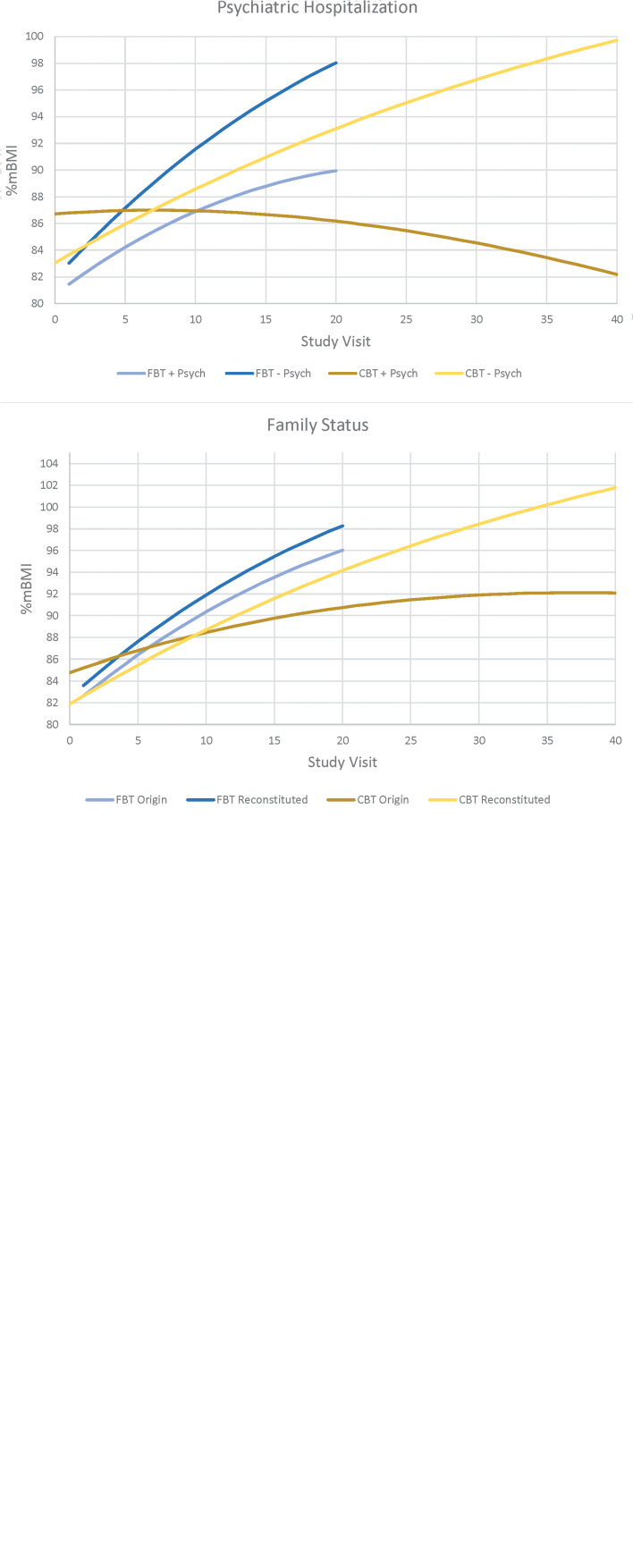
Potential treatment effect moderators at end-of-treatment for the lower weight cohort.

Four baseline variables emerged as potential treatment effect moderators of weight gain at EOT for the *lower weight* cohort only; CDI-2 (est. = 0.008, s.e. = 0.003, *p* = 0.020), age (est. = −0.072, s.e. = 0.023, *p* = 0.002), prior hospitalization for a co-existing psychiatric disorder (est. = −0.187, s.e. = 0.093, *p* = 0.048), and family status (family of origin/reconstituted family^3^) (est. = −0.247, s.e. = 0.089, *p* = 0.007). Weight gain for FBT patients was faster than for those in CBT-E, with comparable rates of weight gain in FBT regardless of depression level, but significantly poorer weight gain for those in CBT-E with higher levels of depression. Patients gained weight faster in FBT than CBT-E regardless of age, but patients in FBT who were younger had better weight gain than those who were older, whereas patients in CBT-E who were older had better weight gain than those who were younger. Patients without a history of psychiatric hospitalization gained weight in FBT and CBT-E, whereas a positive history of prior psychiatric hospitalization was associated with weight gain in FBT, but weight loss in CBT-E. For both treatments, patients living within their family of origin had less weight gain than those living in reconstituted families, but the difference was more pronounced in CBT-E (poorer weight gain if living in family of origin) (Fig. 4). No variables emerged as significant treatment effect moderators of EDE/Q at EOT or follow-up.

### Non-specific predictors of primary outcome

Non-specific predictors of weight gain in the *lower weight* cohort included CBCL externalizing (est. = −0.009, s.e. = 0.004, *p* = 0.025), a co-occurring DSM psychiatric disorder (est. = 0.128, s.e. = 0.043, *p* = 0.004), a history of abuse (est. = −0.184, s.e. = 0.049, *p* < 0.001), and a history of previous mental health treatment (est. = 0.159, s.e. = 0.049, *p* = 0.001). A higher rate of weight gain was achieved for those with lesser externalizing problems, no co-occurring psychiatric disorder, no prior mental health treatment, and no history of abuse. History of abuse was also a non-specific predictor of outcome in the *higher weight* cohort (est. = −0.082, s.e. = 0.038, *p* = 0.032), such that rate of weight gain was poorer for those with a history of abuse. No variables emerged as significant non-specific predictors of EDE/Q at EOT or follow-up.

### Hospitalization during treatment phase

The percentage of patients who were hospitalized during treatment in the full sample was 9.8% (5 of 51) in FBT, and 21.7% (10 of 46) in CBT-E (Fisher's exact *p* = 0.159). In the *lower weight* cohort, the percentage of patients hospitalized during treatment was 19.0% (4 of 21) in FBT, and 43.8% (7 of 16) in CBT-E (Fisher's exact *p* = 0.151), and in the *higher weight* cohort was 3.3% (1 of 30) in FBT and 10.0% (3 of 30) in CBT-E (Fisher's exact *p* = 0.612).

### Mental health treatment during follow-up

The percentage of patients receiving any mental health intervention post-treatment was 9.8% (5 of 51) in FBT and 6.5% (3 of 46) in CBT-E (Fisher's exact *p* = 0.718) for the full sample. In the *lower weight* cohort, the percentage was 19.0% (4 of 21) for FBT, and 18.8% (3 of 16) in CBT-E (Fisher's exact *p* = 1.00), and in the *higher weight* cohort, the percentage was 3.3% (1 of 30) for FBT, and 0% (0 of 30) for CBT-E (Fisher's exact *p* < 1.00).

### Treatment suitability and patient expectancy

Overall, mean *suitability* ratings for FBT were 7.42 (s.d. = 3.10), and 8.38 for CBT-E (s.d. = 2.66). No significant differences between treatments in suitability ratings were found in the overall sample or separately by weight cohort (all *p*s ⩾0.103). Overall, mean *success* ratings for FBT were 7.89 (s.d. = 3.32), and 8.21 for CBT-E (s.d. = 2.11). No significant differences between treatments were found in the overall sample or separately by weight cohort (all *p*s ⩾0.335).

## Discussion

This non-randomized study set out to compare the relative effectiveness of FBT and CBT-E for adolescents with a DSM-5 eating disorder (80% with a restrictive disorder) in terms of change in weight and eating disorder psychopathology at the end of treatment. Treatment groups were divided into two baseline weight cohorts; *lower v. higher weight*. Patients who chose CBT-E over FBT were older, more depressed, had been ill for longer, reported greater clinical impairment, and a greater portion had a history of prior mental health treatment. For those in the *higher weight* cohort, patients whose families chose CBT-E had higher anxiety, were more likely to have a co-occurring psychiatric disorder, as well as a history of prior psychiatric hospitalization. For those in the *lower weight* cohort, families of the most underweight patients were more likely to have chosen FBT.

Regardless of weight cohort, FBT was more efficient than CBT-E in terms of the slope of weight gain from baseline to the EOT. However, this was no longer the case at either the 6- or 12-month follow-up. Initial more gradual weight gains achieved by CBT-E compared to FBT at EOT may be partly due to distinct strategies used to achieve weight gain across these two treatments. In CBT-E, weight gain (when indicated) is addressed after 4 weeks of treatment, and only when patients reach the conclusion that they need to attend to their low weight (Dalle Grave & Calugi, 2020). In contrast, weight gain in FBT (when indicated) is addressed at the outset, and weight goals are arguably higher than in CBT-E, while parents are supported to drive this agenda (Lock & Le Grange, 2012). That said, for a substantial minority of patients in the *higher weight* cohort (~22%), weight gain was not a treatment goal. Therefore, relative effectiveness was defined in terms of weight gain and/or improvement in eating disorders psychopathology. In this domain, both treatments demonstrated improvements in the EDE/Q Global Score with no significant differences across time.

In terms of the secondary outcomes (controlling for baseline differences), the two treatments largely established similar gains across measures of general psychopathology and clinical impairment. That said, and for the *higher weight* cohort only, parents in FBT compared to CBT-E noted fewer emotional and behavioural problems in their offspring, as reported via the CBCL, at EOT and 12-month follow-up, suggesting an advantage for FBT over CBT-E in this domain. However, this finding should be tempered given the noted differences in baseline profile in that older and more unwell patients' parents opted for CBT-E rather than FBT. Albeit speculatively, it seems that parents considered an individual therapy rather than a family-based one to be more appropriate when their offspring was older and more unwell.

For the *lower weight* cohort only, four baseline variables were identified as potential moderators of treatment outcome at EOT, i.e., depression, age, prior psychiatric hospitalization, and family status. While tentative, it would seem appropriate to recommend CBT-E rather than FBT for *lower weight* patients who present with either lower levels of depression, are older, not living in their family of origin, or have no previous psychiatric hospitalizations. A similar number of baseline variables were identified as non-specific predictors; CBCL, comorbid psychiatric diagnosis, a history of abuse, and a history of prior mental health treatment. That is, for the *lower weight* cohort, and regardless of treatment modality, higher rates of weight gain were achieved for those with a lower CBCL (internalization), and an absence of a comorbid psychiatric history, abuse, or prior mental health treatment. For the *higher weight* cohort, the absence of a history of abuse predicted higher rates of weight gain, irrespective of whether the participants received FBT or CBT-E. While this study allowed for only explorative moderator analyses, it nevertheless adds to our limited capacity to better match patients with one treatment rather than another (c.f. Le Grange et al., 2012, 2016; Madden et al., 2015).

With a broader lens, and consistent with recent RCTs setting a similar albeit low bar for treatment completion (Eisler et al., 2016; Le Grange et al., 2016), 28% of participants did not complete 50% or more of the prescribed dose. Of note, and for the *higher weight* cohort only, the majority of participants in CBT-E (93%) met this threshold, whereas half of those in FBT failed to do so. Keeping in mind that patients in this cohort were, on average, older than those in the *lower weight* cohort, prompts the question whether an individual treatment (CBT-E) is perceived as a better fit than one in which parents take a firm lead (FBT). While our treatment completion data seem to support this hypothesis, it is not borne out by our *treatment suitability and patient expectancy* findings which showed no difference between CBT-E and FBT.

Largely in keeping with rates in recent RCTs (Agras et al., 2014; Le Grange et al., 2016; Lock et al., 2010), there were no differences across weight cohort and treatment modality in terms of hospitalization during treatment (15%); seeking mental health treatment during the follow-up period (10%); or participant report of perceived expectations and suitability of treatment. Taken together, these findings seem to indicate that, outside of initial weight gain, FBT and CBT-E are on par in terms of most desired clinical outcomes.

Some limitations to this effectiveness study should be considered. First, we did not conduct an *a priori* power calculation to guide recruitment efforts. However, our robust findings in terms of the primary outcome measures are reassuring that we were adequately powered to detect clinically meaningful differences between treatment modalities. Second, and by design, participants were not randomly allocated to either FBT or CBT-E; instead families chose between these two treatments. While this design likely contributed to baseline differences between treatment groups, the treatment assignment method is consistent with the ‘real-world’ environment where family/patient preference is accounted for, which we were hoping to emulate in order to accurately measure treatment effectiveness. Third, compliance with post-baseline assessments was less than optimal, and although this may be due to a ‘real-world’ clinical environment as opposed to the expected rigor of an efficacy trial, our findings at follow-up should nevertheless be considered against this reality. That said, retention rates for the primary outcomes at 12-month follow-up were quite similar to the most recently published pragmatic RCT in this domain (c.f., Eisler et al., 2016). Fourth, diversity in our sample was limited and should be taken into account when considering to whom these findings might apply. Some significant strengths ought to be noted. Chief among these are expert training and regular supervision provided to clinicians, and the capacity to employ a research assistant, although not blinded, to conduct gold-standard measures through 12-month follow-up.

The current study underscores the efficiency of FBT to facilitate early weight gain among adolescents who present with a restricting eating disorder. However, in almost all other domains assessed, FBT and CBT-E achieved similar outcomes, and provide additional support for CBT-E as a viable treatment modality for adolescents with an eating disorder. The relative efficacy of FBT and CBT-E for this patient population, however, remains an empirical question that should be tested in a sufficiently powered randomized clinical design.

## Notes

1 ‘Study visit’ refers to the assessment time point at a particular treatment session, that is, Session 0 = Baseline, Session 20 = EOT for FBT and CBT-E (higher weight cohort) and 40 for CBT-E (lower weight cohort).

2 The AN patients were in the higher weight cohort as they were admitted post-baseline assessment, and post discharge weight was utilized to determine weight cohort for outpatient treatment.

3 Reconstituted families are those families experiencing divorce, repartnering, or adoption.

## Supplementary material

For supplementary material accompanying this paper visit https://doi.org/10.1017/S0033291720004407. click here to view supplementary material
