# Supplementary material for: Enhanced cognitive-behavior therapy and family-based treatment for adolescents with an eating disorder: a non-randomized effectiveness trial
Source: Psychol Med. 2020 Dec 3;52(13):2520–30. doi: 10.1017/S0033291720004407 (PMC9647542; doi:10.1017/S0033291720004407)
Supplement: Supplementary file 1 [file S0033291720004407sup.zip › S0033291720004407sup001.docx]

**Enhanced Cognitive-Behavior Therapy (CBT-E)**

Cognitive behavior therapy, or CBT, is a leading evidence based treatment for adolescents and adults with an eating disorder.

Our data indicate that about two-thirds of people who complete treatment make an excellent response. There is no reason why you should not be in this group so long as you throw yourself into treatment and give it priority.

The treatment is a one-to-one talking type of treatment that primarily focuses on what is keeping the eating problem going. It is therefore mainly concerned with the present and the future. It addresses the origins of the problem as needed.

The treatment will be tailored to your specific eating problem and to your needs. You and your therapist will need to become experts on your eating disorder and what is keeping it going. Your family will be involved to help support you in making changes. They will need to attend some of your sessions, especially in the beginning of treatment when you will likely need more help making changes. It is important that you are coming to treatment of your own free will and that you have input into how your family will best assist you.

*(For Patients who are at least 90% of Expected Body Weight)* Treatment will involve 20 sessions over 20 weeks plus one initial assessment session, the first eight sessions being twice a week, the next 10 being weekly, and the last three being at two-week intervals.

(For Patients who are less than 90% of Expected Body Weight) Treatment will involve about 40 sessions over approximately 40 weeks, the first 20 or so sessions being twice a week. Thereafter they will spread out.

It is important that there are as few breaks in treatment as possible. This is because we want to establish what we call “momentum” in which we work from session to session to break into your eating problem. Breaks in treatment are very disruptive as momentum is lost. It is especially important that there are no breaks in the first six weeks and no longer than two-week breaks thereafter. We need to take this into account when thinking when it would be best for your treatment to start.

Each appointment will last just under one hour.

For everyone’s sake it is important that appointments start and end on time. Your therapist will make sure he or she is ready at the due time and we request that you do the same. It is a very good idea to arrive a little time in advance – say 10 to 15 minutes beforehand. This will give you an opportunity to settle down and think over things.

If you are unable to attend a specific appointment, please let us know as soon as possible so that we can reschedule the appointment and offer someone else your slot.

You and your therapist will be working together as a team to help overcome your eating problem.

You and your therapist will agree upon specific tasks (or “next steps”) for you to undertake between each session. These tasks are very important and will need to be given priority. It is what you do between sessions that will govern to a large extent how much you benefit from treatment.

It is very important that you make the most of this opportunity to change, otherwise the eating problem is likely to persist.

Treatment will be hard work but it will be worth it. The more you put in, the more you will get out.
