# Supplementary material for: Enhanced cognitive-behavior therapy and family-based treatment for adolescents with an eating disorder: a non-randomized effectiveness trial
Source: Psychol Med. 2020 Dec 3;52(13):2520–30. doi: 10.1017/S0033291720004407 (PMC9647542; doi:10.1017/S0033291720004407)
Supplement: Supplementary file 1 [file S0033291720004407sup.zip › S0033291720004407sup002.docx]

**Family-Based Treatment (FBT)**

Family-based treatment, or FBT, is the leading evidenced based therapy for children and adolescents with an eating disorder.

Our data indicate that about two-thirds of people who complete treatment make an excellent response.

The treatment is a talking type of treatment that involves the whole family and primarily focuses on helping parents play an active and positive role in helping to restore the patient’s weight, then hand control of eating back to the patient as is age appropriate. Toward the end of treatment, it is concerned with developmental issues that may have been impacted by the illness.

The treatment will be tailored to your family member’s specific eating problem and to your needs. You and your family will learn to recognize the behaviors of the illness and of malnutrition while maintaining a positive connection to your family member.

Treatment will involve about 20 sessions over about 6 months. Initially sessions will be once weekly, then every other week.

It is important that all family members attend the sessions, and that there are as few breaks in treatment as possible. This is because we want to establish what we call “momentum” in which we work from session to session to disrupt the illness. Breaks in treatment are very disruptive as momentum is lost. It is especially important that there are no breaks in treatment in the first six weeks. We need to take these factors into account when thinking when it would be best for your treatment to start.

Each appointment will last just under one hour.

For everyone’s sake it is important that appointments start and end on time. Your therapist will make sure he or she is ready at the due time and we request that you do the same. It is a very good idea to arrive a little time in advance – say 10 to 15 minutes beforehand. This will give you an opportunity to settle down and think over things.

If you are unable to attend a specific appointment, please let us know as soon as possible so that we can reschedule the appointment and offer someone else your slot.

You and your family will be working together with your therapist as a team to help overcome the illness.

You and your family will agree to work on specific strategies in order to take an active role in your family member’s recovery. These tasks are very important and will need to be given priority. It is what you do between sessions that will govern to a large extent how much your family benefits from treatment.

Since the risks for your family member are quite high, it is really important that you make the most of this opportunity to learn how to intervene, otherwise the problem is likely to persist.

Treatment will be hard work but it will be worth it. The more you and your family put in, the more you will get out.
